# Identification of novel protein biomarkers and drug targets for functional dyspepsia by integrating human plasma proteome with genome

**DOI:** 10.1097/MD.0000000000044660

**Published:** 2025-09-19

**Authors:** Jiexin Zhou, Shuxian Liu, Zhipeng Jia, Bo Ma, Yanan Luo, Jiayan Wang, Hui Fang, Yiwei Guan, Yuanxiao Li

**Affiliations:** a Department of Pediatrics, Fu Yang People’s Hospital, Fuyang, Anhui, China; b The Second Clinical Medical School, Lanzhou University, Lanzhou, Gansu, China; c Department of Pediatric Gastroenterology, The Second Hospital, Lanzhou University, Lanzhou, Gansu, China.

**Keywords:** drug targets, functional dyspepsia, human plasma proteome with genome, novel protein biomarkers

## Abstract

This study aimed to identify novel therapeutic targets for functional dyspepsia (FD) using Mendelian randomization (MR) and assess the feasibility of candidate drugs for FD treatment. MR analysis was employed to identify cis-acting protein quantitative trait loci (cis-pQTLs) significantly associated with FD. Initial MR analysis was conducted using the TwoSample MR software package. Cochran Q-test and MR-Egger intercept test were applied to evaluate potential heterogeneity and horizontal pleiotropy, respectively. Cis-pQTLs exhibiting consistent effect estimates in the same direction were prioritized. Subsequent analyses included gene ontology enrichment, Kyoto Encyclopedia of Genes and Genomes enrichment, and protein–protein interaction network construction. Potential therapeutic compounds were predicted using the Drug Signatures Database, followed by molecular docking to evaluate their binding affinities with the target protein. The analysis identified 10 cis-pQTLs causally linked to FD. Certain cis-pQTLs were associated with a reduced risk of FD (odds ratio < 1), while others were associated with an increased risk (odds ratio > 1). Specific cis-pQTLs were identified whose corresponding protein levels serve as established diagnostic biomarkers for disease risk and pathological status. Five potential therapeutic drugs for FD were predicted through this integrated approach. Among the candidate drugs, Esculetin demonstrated notable pharmacological activities, including antioxidant and anti-inflammatory effects. Scopoletin exhibited a bidirectional modulatory effect on smooth muscle contraction, suggesting potential benefits in improving gastrointestinal motility. These 2 compounds represent the most promising candidates for FD therapy. This study delineated a rigorous multi-omics integration pipeline to identify potential therapeutic targets and drugs for FD. These findings provide new insights for developing therapeutic agents against FD.

## 1. Introduction

Functional dyspepsia (FD), one of the most common functional gastrointestinal disorders, is defined by chronic or recurrent epigastric symptoms – such as postprandial fullness, early satiation, epigastric pain, or burning – in the absence of structural abnormalities identified through routine clinical evaluation.^[1–3]^ FD is classified into 2 subtypes based on symptoms: postprandial distress syndrome and epigastric pain syndrome. Uninvestigated dyspepsia affects up to approximately 20% of the general population. Although a minority of these patients frequently seek care in outpatient settings due to persistent or recurrent symptoms,^[4,5]^ FD poses a significant healthcare challenge, substantially impairing patients’ quality of life and imposing a substantial burden on healthcare systems worldwide.

FD has a complex etiology involving an interplay of factors, including gastrointestinal motility disturbances, visceral hypersensitivity, abnormalities in gastric acid secretion, and diminished digestive enzyme activity^[6,7]^ Conventional pharmacotherapy typically targets motility (prokinetics) and acid secretion (acid suppressants); however, treatment responses are frequently suboptimal, accompanied by high rates of symptom recurrence.^[8]^

Proteins are fundamental components of biological regulation and represent primary targets for therapeutic intervention. The blood–brain barrier separates circulating proteins into 2 distinct groups: plasma proteins and cerebrospinal fluid proteins. Plasma proteins play critical roles in diverse biological processes (BPs) and are major druggable targets.^[9]^

Protein quantitative trait loci (pQTL) can be divided into 2 categories: cis-acting protein quantitative trait loci (cis-pQTL) and trans-protein quantitative trait loci (trans-pQTL). Cis-pQTLs are genetic variants located within a fixed physical distance (typically ± 1 Mb) from the coding gene of the regulated protein, directly influencing its expression level. In contrast, trans-pQTLs reside distally (>1 Mb) or on different chromosomes, modulating protein expression via indirect mechanisms such as transcription factors or signaling pathways. Previous studies have shown that selection of cis-pQTL for instrumental variable (IV) can reduce the effects of false positives and pleiotropy.^[10]^ Hence, cis-pQTL are considered to have more direct biological effects on proteins.

FD lacks targeted therapies: Current regimens, including proton pump inhibitors, prokinetics, and antidepressants, primarily target symptom alleviation rather than underlying pathophysiological mechanisms.^[3]^ No single treatment demonstrates consistent efficacy across all FD subtypes.

Multiplex proteomic assays enable simultaneous quantification of multiple circulating protein biomarkers with potential clinical relevance to disease pathogenesis. However, no cohort study to date has investigated associations between circulating proteins and FD. Mendelian randomization (MR) is a statistical method that uses genetic variation as an IV to infer causal relationships between an exposure and an outcome.^[11]^ Compared to traditional observational studies, MR analysis reduces both confounding and reverse causation. This is because alleles are randomly allocated at conception during meiosis, providing stronger evidence for causal inference. Despite a growing body of research on FD treatments, the exploration of plasma protein drug targets for FD using MR remains absent from the literature.

This study aims to discover novel therapeutic targets for FD. Subsequently, leveraging network pharmacology and molecular docking methodologies, we will identify potential drug candidates capable of modulating these targets, thereby evaluating their feasibility for drug repurposing or development.

## 2. Material and methods

### 2.1. Ethical approval

This study utilized public-domain summary-level data, with all original studies having obtained prior ethical approvals from their respective institutional ethics committees.

### 2.2. Data sources

The plasma cis-pQTLs data are from the large scale integrative study by Ferkingstad et al,^[12]^ who provided 4907 cis-pQTLs data from 35,559 Icelanders. We selected cis-pQTLs according to the following criteria: cis-pQTLs show a genome-wide significant association (*P* < 5 × 10⁻⁸). Meet the independence assumption (linkage disequilibrium [LD] clustering *r*² = 0.0001). cis-pQTLs span the corresponding protein-coding sequence within a 1000 kb window. cis-pQTLs are strong IVs (F-statistic >10). Exclude single-nucleotide polymorphisms (SNPs) with palindromes and SNPs with missing data. Finally, 31,315 SNPs of 3131 cis-pQTLs were included (Table S1, Supplemental Digital Content, https://links.lww.com/MD/Q53). The FD data are from the FinnGen database.^[13]^ The FinnGen study is a large-scale genomics initiative that has analyzed over 500,000 Finnish biobank samples and correlated genetic variation with health data to understand disease mechanisms and predispositions. The project is a collaboration between research organizations and biobanks within Finland and international industry partners.

### 2.3. MR analysis

In the main MR analysis, cis-pQTLs were used as IVs, and FD was used as the outcome. When there is only 1 cis-pQTL as an IV, the Wald ratio method was used to estimate the causal effect.^[14]^ If there are 2 or more IVs available, 5 methods including MR Egger, weighted median, inverse variance weighted, simple mode, and weighted mode were used.^[15]^ The statistical results are presented in the form of odds ratios and 95% confidence intervals. We considered a *P*-value < .05 as the nominal significance threshold. Genetic instrument selection criteria were applied as follows: SNP-protein association screening: Genetic variants significantly associated with plasma protein levels (*P* < 5 × 10^−8^) were included as candidate instruments. MHC region exclusion: variants and proteins within the major histocompatibility complex (chr6:25.5–34.0 Mb) were excluded due to complex LD patterns. LD clumping for independence: Independent cis-pQTL were identified by LD-based clumping (*r*^2^ < 0.001) per protein. Instrument strength evaluation: the explanatory power of each instrument was quantified using: variance proportion (*R*^2^ = 2 × EAF × (1 − EAF) × beta^2^) F-statistic F = *R*^2^ × (N − 2)/(1 − *R*^2^)). For proteins measured across multiple studies, the instance with the maximal cumulative *R*^2^ was retained.

### 2.4. Visualization of MR results

In this study, R-language software was used to visualize the genetic associations between cis-pQTLs and FD. The “reshape2” (V.1.4.4), “circlize” (V.0.4.16), and “ComplexHeatmap” (V.2.22.0) packages were used to draw Circos plots. The clustering diagrams are located inside the circular plots, and the names of cis-pQTLs are located outside the circular plots. The “grid” (V.4.4.1), “ggdendro” (V.1.4.4), and “forestploter” (V.1.12) packages were used to draw forest plots of MR results.

### 2.5. Identification of differentially expressed cis-pQTLs

The “ggplot2” (V.3.5.1), “ggrepel” (V.0.9.6) packages in R software were applied to perform differential expression analysis of cis-pQTLs on the results of MR. A *P*-value < .05 was used as the cut-off point for differential cis-pQTL analysis, and the differential expression patterns of cis-pQTLs were shown using volcano plots.

### 2.6. Identification of cis-pQTLs related to FD

The “ggplot2” (V.3.5.1), “tidyverse” (V.1.3.1), and “ggMplot” (V.4.5.1) packages in R software were applied to identify cis-pQTLs related to FD, and Manhattan plots were drawn to display the specific names of cis-pQTLs related to FD.

### 2.7. Enrichment analysis

To explore the functional characteristics and biological relevance of pre-determined future druggable genes, the names of cis-pQTL proteins related to FD were extracted. The names of cis-pQTLs were converted into gene IDs, and genes with sNA gene IDs were removed. The “clusterProfiler” (V.4.10.1)^[16]^ package in R software was used for gene ontology (GO) and Kyoto Encyclopedia of Genes and Genomes (KEGG) enrichment studies. GO analysis categorizes gene functions into 3 domains: molecular function, BP, and cellular component. The KEGG database integrates genomic information to systematically annotate gene functions, pathways, and biological systems.

### 2.8. Construction of protein–protein interaction (PPI) networks

The PPI network can visually display the relationships among the interactions of proteins of important drug genes. We used STRING (https://string-db.org/) to construct the PPI network, with a confidence score threshold of 0.15 as the minimum interaction score required, and all other parameters remained at their default settings.^[17]^

### 2.9. Screening of hub genes

The cytoHubba-v0.1 plugin of Cytoscape 3.10.3 was used to analyze the PPI network file string_interactions_short.tsv, and the top 10 hub genes were selected by screening at the degree level.

### 2.10. Prediction of candidate drugs

The Drug Signatures Database (DSigDB, https://dsigdb.tanlab.org/DSigDBv1.0/)^[18]^ is a fairly large database with 22,527 gene sets and 17,389 unique compounds, spanning 19,531 genes. We uploaded the previously identified important drug-targetable genes to DSigDB to predict candidate drugs and evaluate the pharmacological activities of target genes.

### 2.11. Drug enrichment analysis

We used the screened hub genes and the drug–gene relationship files downloaded from DSigDB, and used R software packages “clusterProfiler” (V.4.10.1), “org.Hs.eg.db” (V.3.20.0), “enrichplot” (V.1.266), and “ggplot2” (V.3.5.1) for drug enrichment analysis.

### 2.12. Molecular docking

**Figure 1.**
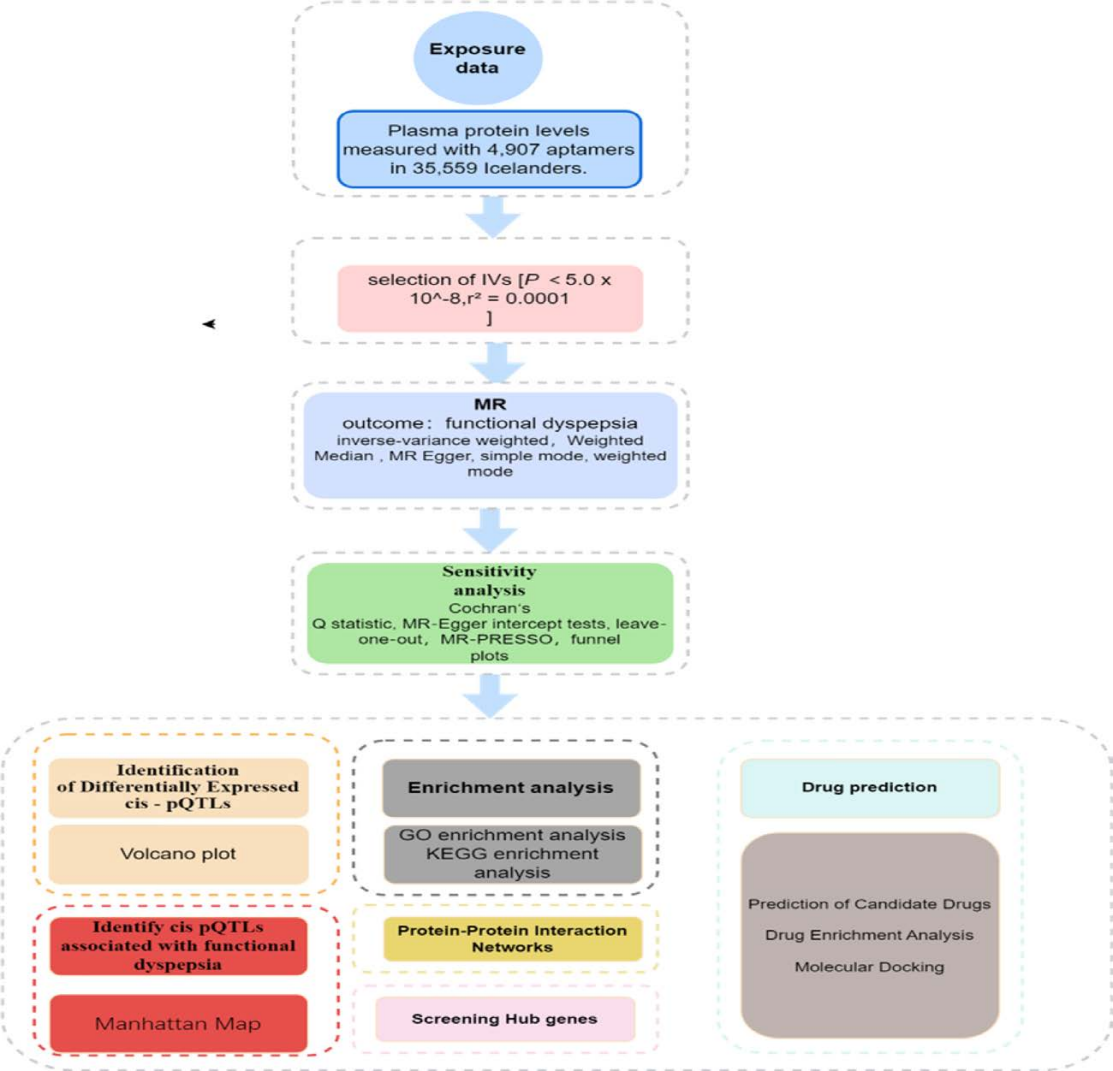
Overview of this MR study. cis-pQTLs = cis-acting protein quantitative trait loci, GO = gene ontology, IV = instrumental variable, KEGG = Kyoto Encyclopedia of Genes and Genomes, MR = Mendelian randomization.

We performed molecular docking to evaluate the binding energy and interaction modes between candidate drugs and their targets. By identifying ligands that exhibit high binding affinity and beneficial interaction modes, we were able to optimize this as a suggestive result. The TwoSampleMR software package (V.0.6.8) in R software (V.4.4.1) was used for preliminary MR analysis. To make our research results robust and reliable, we screened cis-pQTLs with consistent odds ratio value directions from the 5 methods. Cochran Q-test and MR Egger intercept test were used to evaluate potential heterogeneity and horizontal pleiotropy (*P* > .05 indicates no heterogeneity or horizontal pleiotropy). First, additional experimental validations were considered for drug targets, and the design of potential candidate drugs was refined. The drug structure data were sourced from the PubChem compound database (https://pubchem.ncbi.nlm.nih.gov/), downloaded in SDF format. The protein structure data were from the Protein Data Bank (http://www.rcsb.org/). The computer-based protein-ligand docking website CD-dock2 was used for molecular docking of the top 5 important drugs and the proteins encoded by their respective target genes (https://cadd.labshare.cn/cb-dock2/index.php^[19,20]^), and the final structures of 5 proteins and 5 drugs were obtained. The overall study design is illustrated in Figure 1.

## 3. Results

### 3.1. Identification of FD-associated proteins

**Figure 2.**
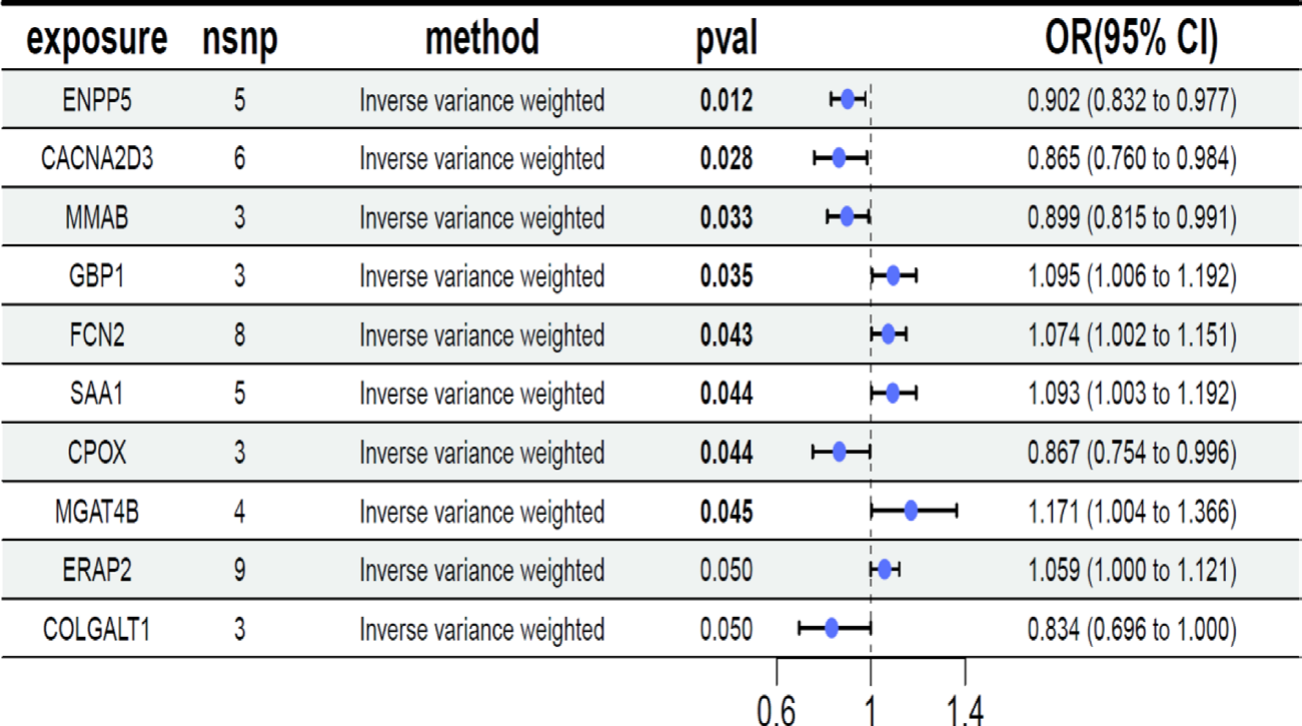
The forest plot of Mendelian randomization result. MR analyses of the effect of proteins on FD outcomes. The squares are the causal estimates on the OR scale, and the whiskers represent the 95% confidence intervals for these ORs.nsnp: number of SNPs used for the estimation of the causal effects in this plot. pval were determined from the inverse-variance-weighted two-sample MR method. FD = functional dyspepsia, MR = Mendelian randomization, OR = odds ratio, SNPs = single-nucleotide polymorphisms.

**Figure 3.**
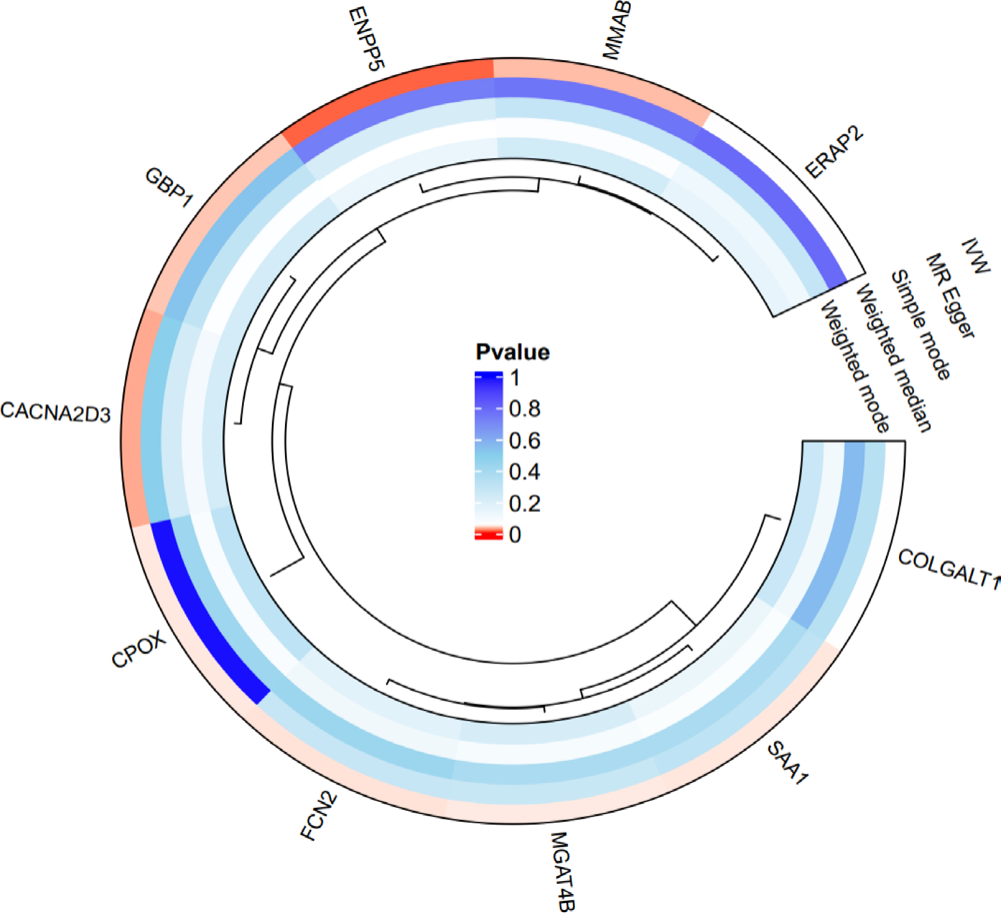
The circos plot of mendelian randomization result. The clustering diagrams are located inside the circular plots, and the names of cis-pQTLs are located outside the circular plots. We performed Mendelian randomization analysis for each protein using 5 distinct methods. The color coding in the visualizations represents the range of *P*-values obtained. Specifically, associations achieving statistical significance (*P*-value < .05) are highlighted in red. cis-pQTLs = cis-acting protein quantitative trait loci.

**Figure 4.**
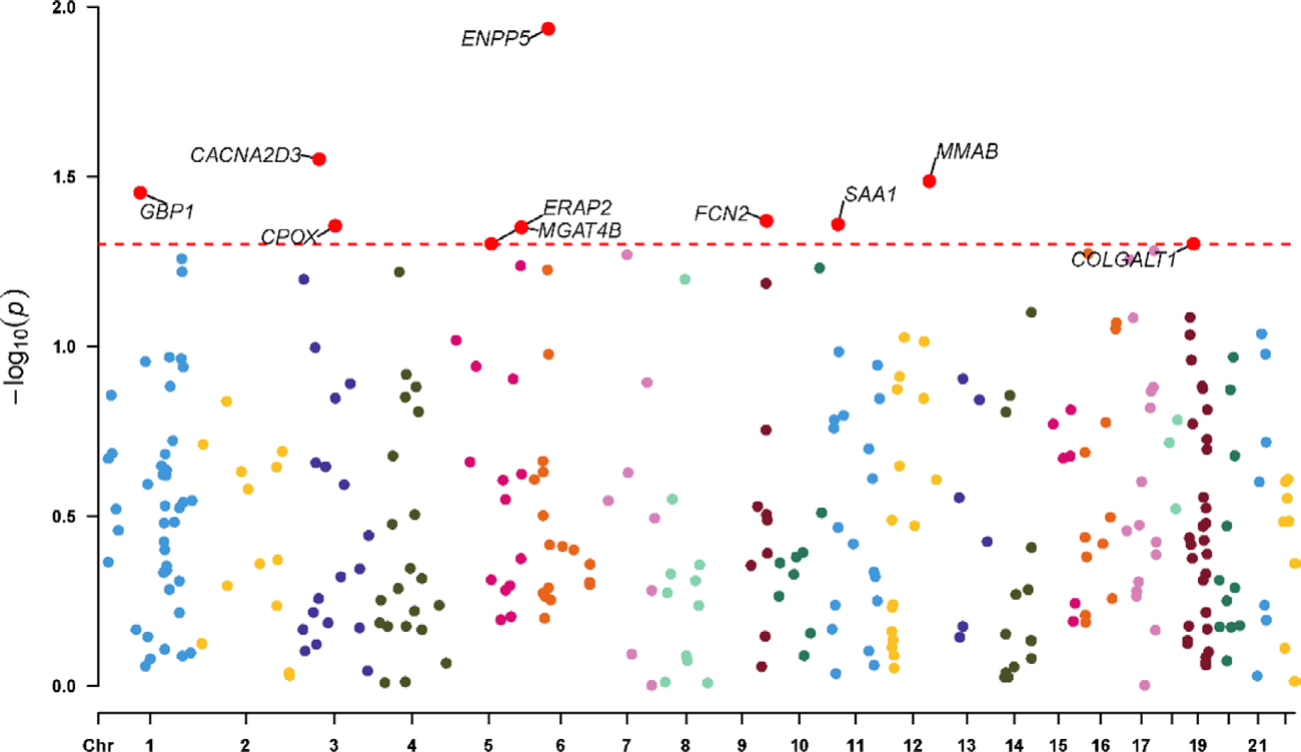
The Manhattan plot of mendelian randomization result. Vertical axis: the *P*-value for each cis-pQTL, ranging from 0 to 1. Lower *P*-values indicate stronger statistical evidence. Typically, the negative logarithm of the *P*-value is plotted (−log_10_(P)). This transformation ensures that smaller *P*-values correspond to higher points on the axis, making them visually distinct and easier to identify. Horizontal axis:The chromosomal position of each SNP. Horizontal line:This represents the significance threshold line, commonly set at *P*-value < 0.05 (or a genome-wide significance level after correction). It allows for immediate visual interpretation of which points reach statistical significance. cis-pQTLs = cis-acting protein quantitative trait loci, SNP = single-nucleotide polymorphism.

As cis-pQTLs were considered to have a more direct and specific biological effect on the protein (compared to trans-pQTLs), we first performed MR analyses using only cis-pQTLs as IVs.10 cis-pQTLs were tested for causal relationships with FD outcomes (Figs. 2–4 and Table S2, Supplemental Digital Content, https://links.lww.com/MD/Q53).

Results of sensitivity analyses confirmed the robustness of the primary MR analyses. There was no evidence for heterogeneity in the association of any of the 10 proteins in Table S3, Supplemental Digital Content, https://links.lww.com/MD/Q53 as measured by Cochran Q statistics (PQ-stat > 0.05), and no indication that the IVs had horizontal pleiotropy as assessed by MR-Egger intercept (*P* Egger-intercept > s.05; Table S4, Supplemental Digital Content, https://links.lww.com/MD/Q53).

### 3.2. Identification of differentially expressed cis-pQTLs in FD

**Figure 5.**
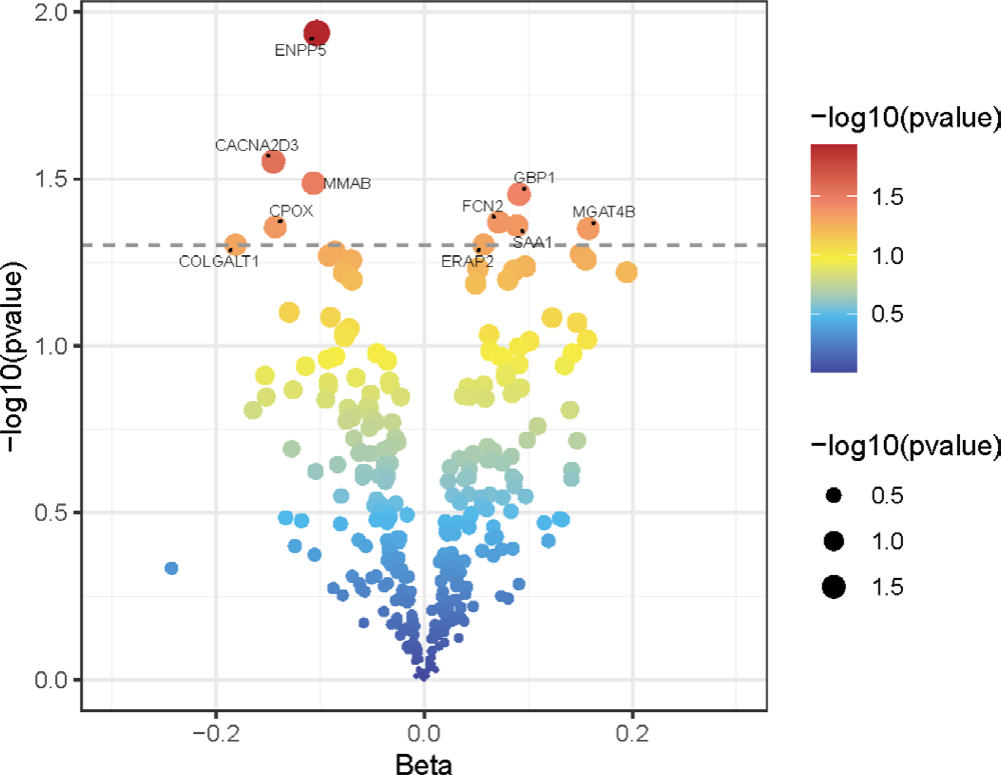
Volcano plot showing results of differentially expressed cis-pQTLs in FD. *X*-axis (horizontal axis): represents the log₂ fold change in expression levels for the cis-pQTLs. Positive values indicate upregulation of gene expression, while negative values indicate downregulation. *Y*-axis (vertical axis): displays the statistical significance of the difference, represented as the negative base-10 logarithm of the *P*-value (−log₁₀(*P*-value)). Interpretation: points positioned farther to the left or right on the *X*-axis correspond to larger magnitude fold changes (greater upregulation or downregulation, respectively). Points located higher on the *Y*-axis represent results with higher statistical significance (smaller *P*-values).

The volcano plot, a core visual tool in differential expression protein analysis, integrates fold change and statistical significance (*P*-value) to display differences in cis-pQTL-associated protein expression in FD (Fig. 5).

### 3.3. Genes and functions

**Figure 6.**
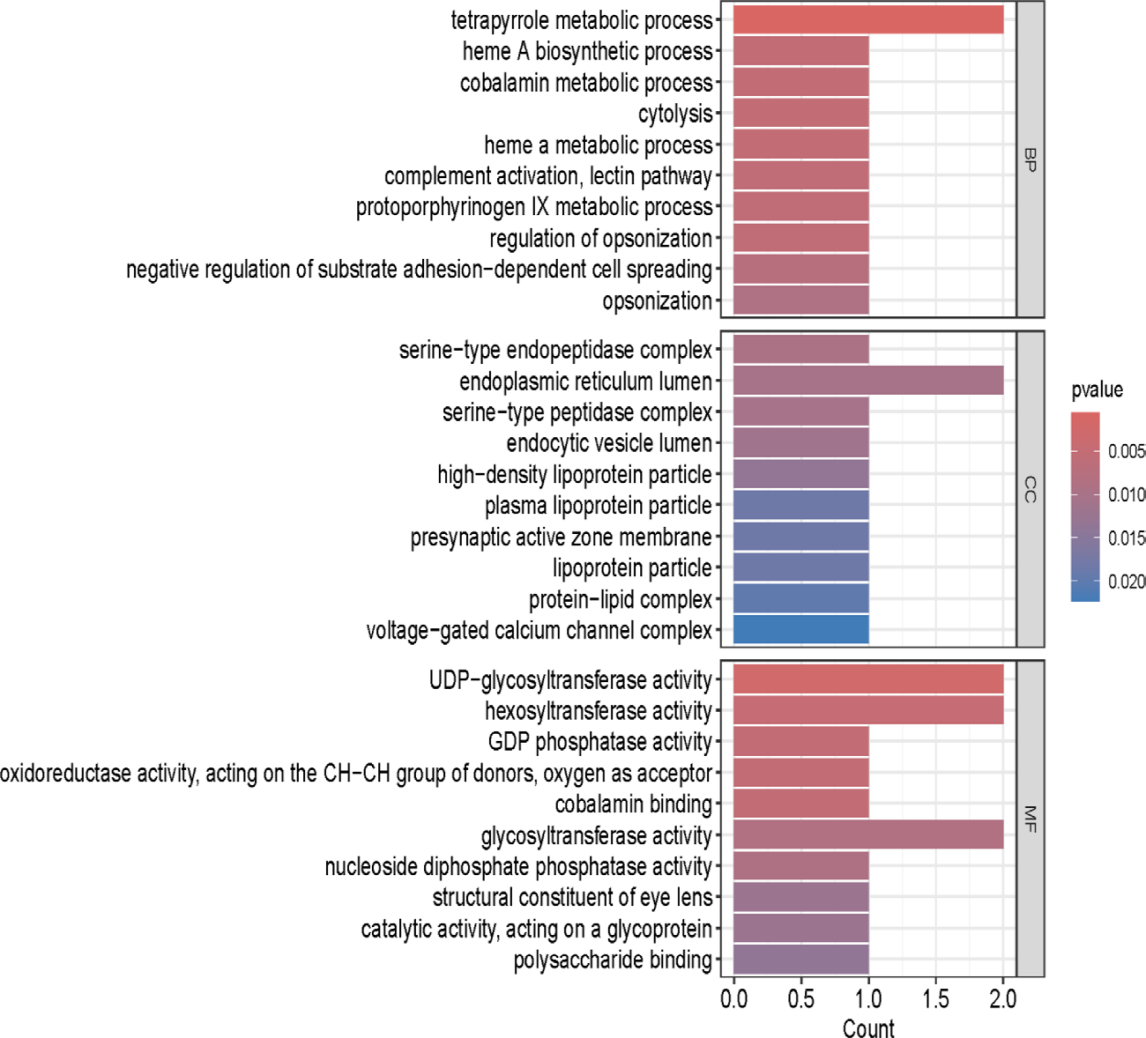
GO enrichment analysis for differentially expressed cis-pQTLs related gene in FD. The figure illustrates the number of genes enriched in various functional categories.The height of each bar corresponds to the number of genes enriched in a specific pathway. The color of the bar indicates the enrichment *P*-value; a smaller *P*-value denotes greater significance. cis-pQTLs = cis-acting protein quantitative trait loci, FD = functional dyspepsia, GO = gene ontology.

**Figure 7.**
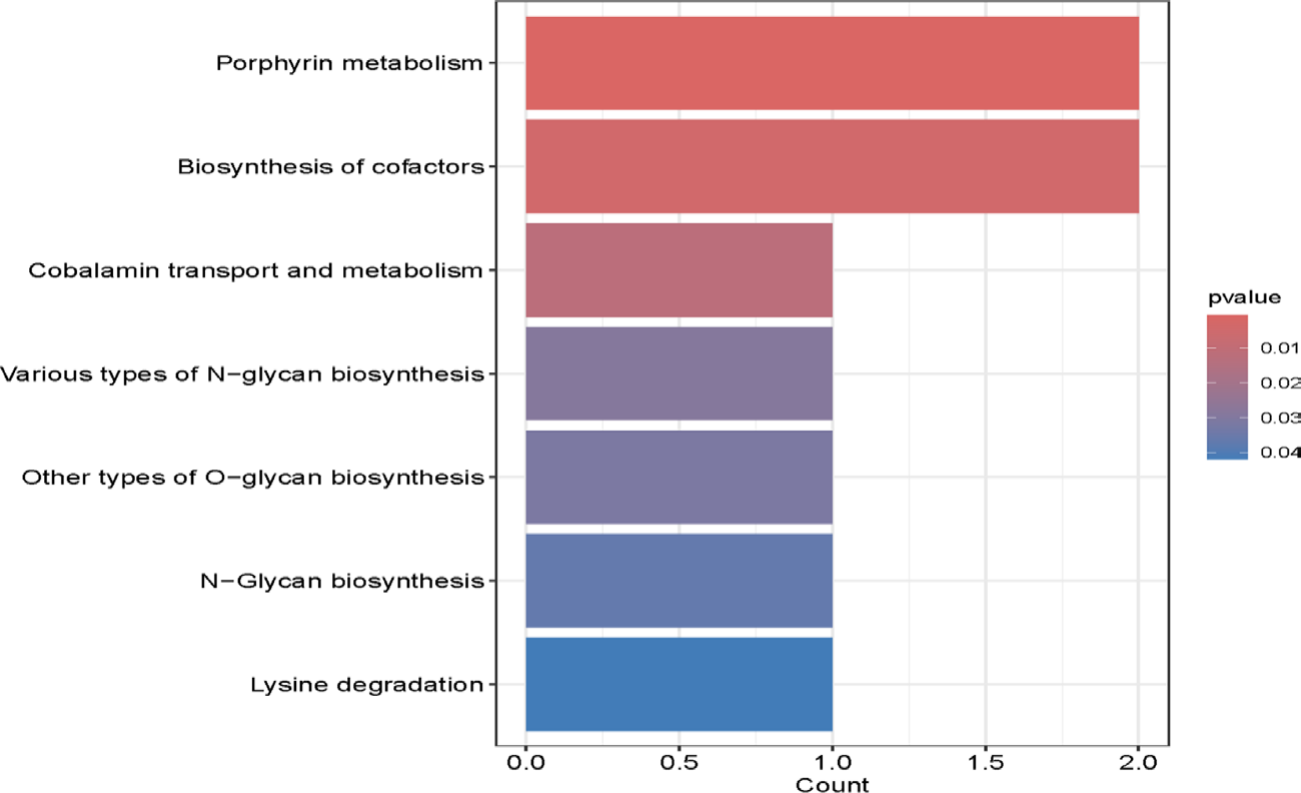
KEGG enrichment analysis for differentially expressed cis-pQTLs related gene in FD. The figure illustrates the number of genes enriched in various functional categories.The height of each bar corresponds to the number of genes enriched in a specific pathway. The color of the bar indicates the enrichment *P*-value; a smaller *P*-value denotes greater significance. cis-pQTLs = cis-acting protein quantitative trait loci, FD = functional dyspepsia, KEGG = Kyoto Encyclopedia of Genes and Genomes.

We conduct GO enrichment analysis for differentially expressed cis-pQTLs in FD across 3 ontological categories: cellular component, molecular function, and BP (Fig. 6). Concurrently, we conducted KEGG enrichment analysis of signaling pathways associated with cis-pQTL-linked genes in functional dyskinesia (Fig. 7).

### 3.4. Construction of PPI networks

**Figure 8.**
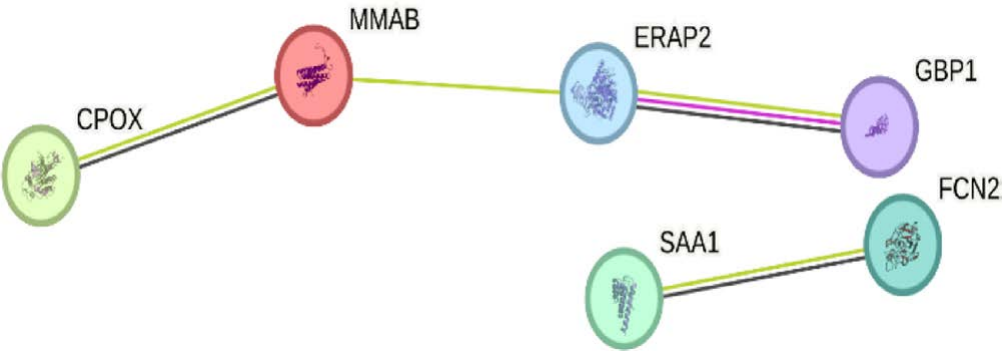
The interaction network of key target proteins. The line represents the type of interaction; The figure contains 6 nodes and 8 edges.

**Figure 9.**
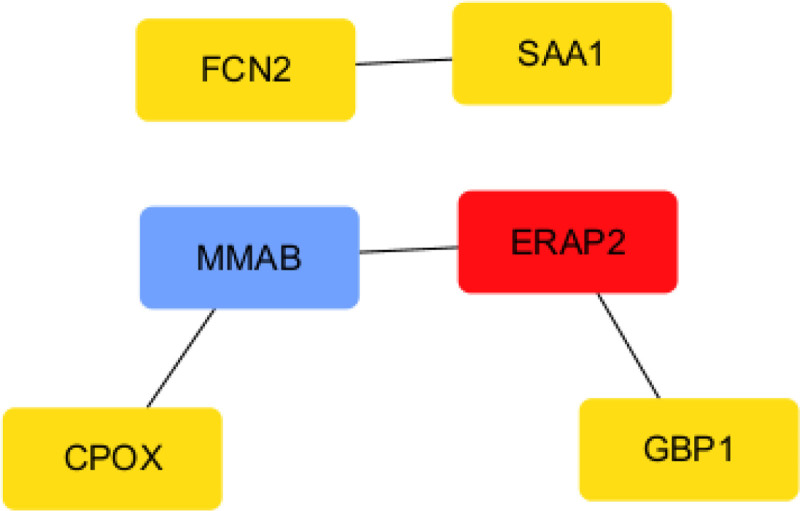
The top six hub genes of PPI network. Connecting lines indicate interactions or associations among the genes. Core genes possess higher network connectivity and are highlighted through increased node size and color saturation.

Using the STRING database, we input the 11 common targets to generate PPI networks. The resulting network comprised 6 nodes and 8 edges. Subsequent analysis using the cytoHubba module identified a core interaction network among these 6 targets. These 6 targets represent key players and provide valuable focal points for subsequent research (Figs. 8 and 9).

### 3.5. Drug enrichment analysis

**Figure 10.**
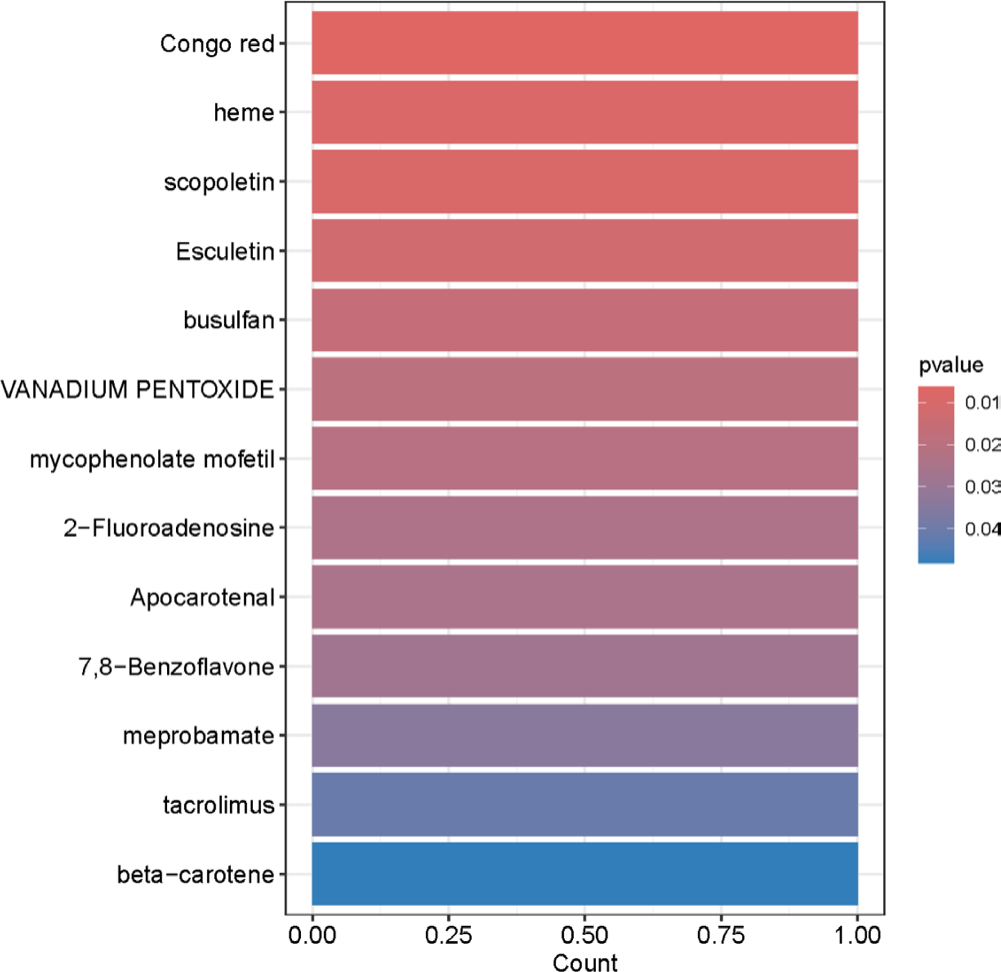
Drug enrichment analysis for differentially expressed cis-pQTLs related gene in FD. In this figure, the vertical axis displays the drug names, the horizontal axis represents the number of genes enriched for each drug, and the column color indicates the statistical significance of the enrichment. Darker red indicates greater significance of core network gene enrichment for the corresponding drug. cis-pQTLs = cis-acting protein quantitative trait loci, FD = functional dyspepsia.

Drug enrichment analysis enables the identification of pharmacological compounds that show significant associations with hub genes in the network (Fig. 10, Table S5, Supplemental Digital Content, https://links.lww.com/MD/Q53).

### 3.6. Molecular docking

**Figure 11.**
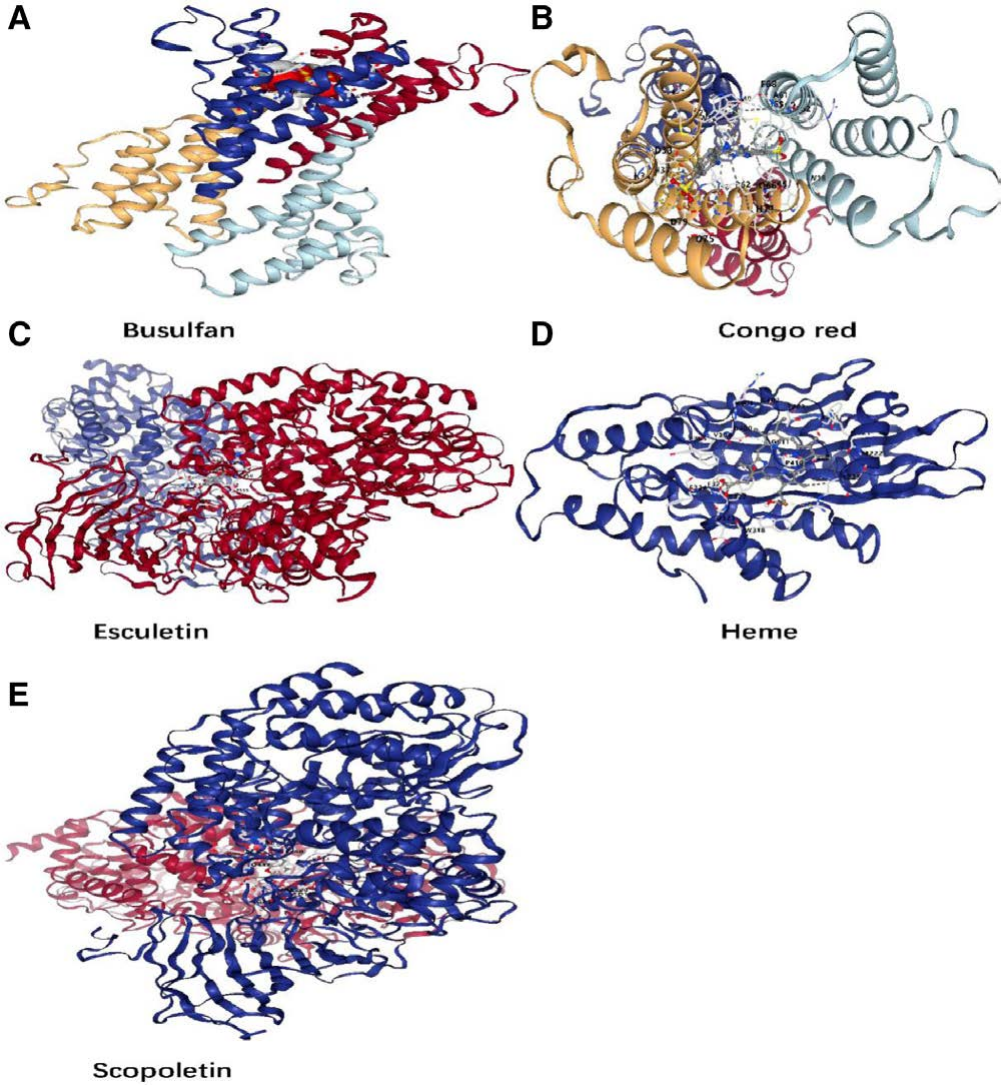
Molecular docking of key target protein and active constituents. (A) Busulfan binding with SAA1. (B) Congo red binding with SAA1. (C) Esculetin binding with ERAP2. (D) Heme binding with CPOX. (E) Scopoletin binding with ERAP2.

We employed CB-DOCK2 to analyze the binding energies of interactions between 5 candidate drugs and their corresponding target proteins. Successful docking poses were obtained for all 5 drug-protein pairs. Figure 11 details the interacting amino acid residues and hydrogen bond lengths identified in these docked complexes.

## 4. Discussion

This study accomplished its dual aims: identifying novel plasma protein targets with causal roles in FD via MR, and assessing repurposable drugs against these targets. We identified 10 causal cis-pQTLs significantly associated with FD risk. Notably, Esculetin and Scopoletin were prioritized as mechanistically supported therapeutic candidates through target binding validation, demonstrating our pipeline’s efficacy in translating target discovery into druggable interventions.

Specific plasma protein levels serve as the most widely utilized diagnostic indicators for assessing disease risk and pathological states. Elucidating the genetic architecture and multifactorial determinants of the plasma proteome is critical to advancing biomarker discovery and facilitating the development of novel therapeutics.^[21–23]^

Treatment with the antidepressant escitalopram upregulates hippocampal ENPP5 expression, concurrently ameliorating FD-like symptoms including intestinal barrier restoration and depressive behaviors. This suggests that ENPP5 may modulate FD pathogenesis via neuro-immune crosstalk^.[24]^ The CACNA2D3 gene, located at chromosome 3p21.1-p14.3, encodes the auxiliary α2δ3 subunit of voltage-gated calcium channels. Dysfunction of the N-type calcium channel (Cav2.2, encoded by CACNA1B) – whose trafficking and electrophysiological properties are regulated by α2δ3 – may exacerbate visceral hypersensitivity in FD by augmenting nociceptive signaling pathways.^[25]^Dysfunctional MMAB impairs succinyl-CoA production, which consequently disrupts gastric myoelectrical rhythms – particularly abnormal postprandial patterns – and delays gastric emptying.^[26]^GBP1 is a large GTPase induced by interferon Overexpression of GBP1 inhibits organoid growth and stem cell renewal in colon organoids stimulated by IFN-γ, suggesting that GBP1 is a risk factor for FD.^[27]^ In patients with FD, elevated duodenal mucosal permeability facilitates bacterial translocation. Concurrently, dysregulation of FCN2 – a key soluble pattern recognition molecule in the lectin complement pathway – potentiates local inflammation by impairing microbial clearance, positioning FCN2 as a critical contributor to FD pathogenesis.^[28]^ SAA1, an acute-phase reactant predominantly synthesized by hepatocytes, exhibits a positive correlation with disease activity in inflammatory bowel disease. Elevated SAA1 levels may exacerbate intestinal inflammation, potentially contributing to the pathogenesis of FD.^[29]^ As the rate-limiting enzyme in heme synthesis, CPOX deficiency directly causes Hereditary Coproporphyria. Currently, no studies have investigated CPOX activity or plasma porphyrin levels in FD. Currently, no direct evidence exists linking plasma levels of MGAT4B to FD. ERAP2, a zinc-dependent aminopeptidase localized in the endoplasmic reticulum, primarily functions in the MHC class I antigen presentation pathway by trimming N-terminal residues of antigenic peptides. Deficiency in ERAP2 disrupts the processing of self-antigens or pathogen-derived peptides, leading to aberrant MHC-I presentation. This dysregulation activates local T-cell responses and promotes the release of pro-inflammatory cytokines, potentially contributing to the pathogenesis of FD.^[30]^ COLGALT1 is a key enzyme responsible for posttranslational collagen modification. It catalyzes the galactosylation of hydroxylysine residues via β1-O-linkage, thereby stabilizing the triple-helical structure of collagen and enhancing tissue integrity. As a critical determinant of submucosal matrix stability, COLGALT1 may function as a protective factor against FD by preserving gastrointestinal structural integrity and mitigating visceral hypersensitivity.^[31]^

Potential therapeutic compounds were predicted using DSigDB, followed by molecular docking to evaluate their binding affinities with the target protein. Congo red, an aniline-derived azo dye, specifically binds to the β-pleated sheet conformation of amyloid proteins and exhibits characteristic “apple-green” birefringence under polarized light microscopy, establishing it as the gold-standard diagnostic tool for systemic amyloidosis.^[32]^ Analogues of Congo red (e.g., CR-1-41) may inhibit amyloid aggregation by enhancing proteasomal degradation of misfolded proteins. Their therapeutic potential warrants investigation for intervening in mucosal amyloid deposits – if present – in patients with FD, potentially addressing FD pathology linked to protein misfolding and visceral hypersensitivity. However, it is essential to first confirm the presence of relevant pathological features in FD. This study proposes heme as a potential therapeutic target for FD; however, current research has not yet established heme levels or HMOX1 expression in the gastric/duodenal mucosa of FD patients. This critical gap requires validation through mass spectrometry or immunohistochemical analysis of biopsy tissues. Scopoletin, a natural coumarin derivative, exhibits a bidirectional modulatory capacity on smooth muscle contraction.^[33]^ At low doses, it stimulates muscarinic M3 receptor signaling or inhibits acetylcholinesterase activity, thereby elevating acetylcholine bioavailability. This enhances gastrointestinal propulsive motility and may ameliorate pathophysiological features of FD.^[34]^ Esculetin, a natural coumarin derivative primarily extracted from the bark of Fraxinus rhynchophylla Hance, exhibits a broad spectrum of pharmacological properties. Owing to its multifaceted bioactivities – including antioxidative, anti-inflammatory, antiapoptotic, anticancer, antidiabetic, neuroprotective, cardiovascular protective, and antibacterial effects – esculetin demonstrates therapeutic potential for specific disease indications. Emerging evidence supports its applicability in managing cancer, diabetes mellitus, atherosclerosis, Alzheimer disease, Parkinson disease, nonalcoholic fatty liver disease, and other pathologies.^[35]^ Experimental results demonstrated that esculetin treatment mitigated intestinal pathological damage, reduced serum diamine oxidase levels, and ameliorated inflammation, oxidative stress, and apoptosis while promoting autophagy in intestinal ischemia-reperfusion rats.^[36]^ A study suggests that esculetin exerts therapeutic effects on ulcerative colitis potentially via the prolactin signaling pathway.^[37]^ These findings elucidate the underlying mechanisms of esculetin in FD treatment, providing novel insights for developing FD therapeutics. The pharmacological activity of busulfan is primarily characterized by its cytotoxic properties, which are mechanistically distinct from the pathophysiological underpinnings of FD, including brain-gut axis dysregulation and gastrointestinal motility disorders.^[38]^ Unlike conventional acid-suppressive or prokinetic targets (e.g., H+/K+-ATPase for PPIs), our novel targets likeCOLGALT1 address fundamental gaps in FD pathophysiology – specifically mucosal barrier disruption and neuro-immune dysregulation. Whereas current therapies offer symptomatic relief, compounds such as Scopoletin demonstrate multi-target efficacy against both motility deficits and visceral hypersensitivity, potentially achieving durable remission. Crucially, MR-derived causal evidence elevates target validation beyond correlative studies dominating existing FD drug development.

However, several limitations of this study warrant consideration. Firstly, the generalizability of our findings may be constrained by the exclusively European ancestry of the analyzed population. Although this homogeneity enhances internal validity, it restricts the extrapolation of results to genetically distinct populations with varying environmental exposures. Consequently, further validation across diverse ancestral backgrounds (e.g., Asian, African, or admixed populations) is essential to establish universal pathophysiological relevance. Secondly, while this investigation focused on plasma protein as systemic biomarkers, their levels cannot be directly extrapolated to local tissue microenvironments. Plasma proteomics offers a noninvasive window into disease mechanisms, yet the gastrointestinal tract – where FD originates – may exhibit heterogeneous protein expression patterns due to compartmentalized pathophysiology. Future studies integrating organ-specific expression profiling (e.g., gastric mucosal proteomics) would provide deeper mechanistic insights. Thirdly, the application of stringent statistical thresholds and evidence grading criteria, though methodologically robust, may inadvertently obscure biologically meaningful signals. Such conservative approaches could lead to 2 limitations: potential underestimation of causal relationships where proteins exhibit moderate but consistent effects, and exclusion of downstream effector proteins in pathological cascades initiated by prioritized “driver” proteins. For instance, proteins regulated posttranslationally or exhibiting modest effect sizes – yet functionally critical in FD pathogenesis – might be overlooked. Complementary approaches could mitigate this constraint by contextualizing marginal associations within biological networks.

## 5. Conclusion

In summary, this study conducted a MR analysis of cis-pQTL to identify causal biomarkers associated with FD, followed by network pharmacology screening to explore potential therapeutic agents. The rigorous multi-omics integration protocol and high credibility of the findings provide critical guidance for both prevention and precision treatment strategies for FD. Through an in-depth review of the literature, scopoletin and esculetin emerge as promising therapeutic candidates for FD, demonstrating significant potential that warrants further experimental validation and clinical investigation.

## Acknowledgments

This research was funded by the Cuiying Scientific Training Program for Undergraduates of Lanzhou University Second Hospital (CYXZ2025-36), Student Entrepreneurship and Innovation Action Plan of Lanzhou University (20040050147 and 20250050021), Taishun County Science and Technology Plan Project(2025-04), and Guided Science and Technology Plan Project of Chengguan District, Lanzhou (2025): Social Development Program.

## Author contributions

**Conceptualization:** Yuanxiao Li.

**Data curation:** Jiexin Zhou.

**Funding acquisition:** Yanan Luo, Jiayan Wang.

**Investigation:** Yanan Luo, Jiayan Wang.

**Methodology:** Jiexin Zhou, Shuxian Liu.

**Resources:** Hui Fang, Yiwei Guan.

**Writing – original draft:** Zhipeng Jia, Bo Ma, Yanan Luo.

**Writing – review & editing:** Jiexin Zhou, Shuxian Liu.

## Supplementary Material


